# Enhancing cultural sensitivity in the implementation of the Fertility Quality of Life Tool in Sudan: a science diplomacy perspective

**DOI:** 10.3389/fpubh.2024.1375643

**Published:** 2024-08-21

**Authors:** Rasha R. Bayoumi, Emily Koert, Jacky Boivin, Margaret McConnell, Betelhem Wolde, Fatima Siddiqui, Khalifa Elmusharaf, Kasisomayajula Viswanath

**Affiliations:** ^1^School of Psychology, University of Birmingham Dubai, Dubai, United Arab Emirates; ^2^Department of Educational and Counselling Psychology and Special Education, University of British Columbia, Vancouver, BC, Canada; ^3^Cardiff Fertility Studies Research Group, School of Psychology, Cardiff University, Cardiff, Wales, United Kingdom; ^4^Department of Global Health and Population, Harvard T H Chan School of Public Health, Boston, MA, United States; ^5^School of Public Health, University of Birmingham Dubai, Dubai, United Arab Emirates

**Keywords:** infertility, FertiQoL, quality of life, cultural considerations, methodological considerations, Sudan, science diplomacy, Global South

## Abstract

**Background:**

Infertility is a global health challenge impacting quality of life, particularly in low and middle-income countries such as Sudan. The Fertility Quality of Life (FertiQoL) tool, a standardized questionnaire, is pivotal in assessing fertility-related quality of life. However, existing research on its utility has primarily been conducted in Global North and High-Income Countries, highlighting the need to shift away from neocolonialism to promote truly inclusive research and effective healthcare practices. Science diplomacy, through the adaptation and culturally sensitive implementation of research tools, can serve as a catalyst for addressing health disparities on a global scale. This study aims to assess methodological and cultural considerations that impact the implementation of the FertiQoL tool in Sudan, framed within the context of science diplomacy and neocolonialism. By investigating the challenges and opportunities of utilizing this tool in a non-Western cultural setting, we seek to contribute to the broader discussion on decolonizing global health research.

**Methods:**

Utilizing an explanatory sequential design involving surveys and interviews, we conducted a study in a Sudanese fertility clinic from November 2017 to May 2018. A total of 102 participants were recruited using convenience sampling, providing socio-demographic, medical, and reproductive history data. The Arabic version of FertiQoL was administered, with 20 participants interviewed and 82 surveyed (40 self-administered and 42 provider-administered). We applied descriptive statistics, one-way ANOVA, thematic analysis, and triangulation to explore methodological and cultural nuances.

**Results:**

Most participants were educated women who lived in urban areas. While the ANOVA results revealed no statistically significant differences in FertiQoL scores based on the mode of administration [core score (*F*(2,99) = 1.58, *p* = 0.21, *η*^2^ = 0.03) and domain scores: emotional (*F*(2,99) = 1.85, *p* = 0.16, *η*^2^ = 0.04); mind/body (*F*(2,99) = 1.95, *p* = 0.15, *η*^2^ = 0.04); relational (*F*(2,99) = 0.18, *p* = 0.83, *η*^2^ = 0.04); and social (*F*(2,99) = 1.67, *p* = 0.19, *η*^2^ = 0.03)], qualitative insights unveiled vital cultural considerations. Interpretation challenges related to concepts like hope and jealousy emerged during interviews. Notably, the social domain of FertiQoL was found to inadequately capture the social pressures experienced by infertile individuals in Sudan, underscoring the importance of region-specific research. Despite these challenges, participants perceived FertiQoL as a comprehensive and valuable tool with broader utility beyond assessing fertility-related quality of life.

**Conclusion:**

Our findings emphasize the significance of incorporating cultural sensitivity into the interpretation of FertiQoL scores when implementing it globally. This approach aligns with the principles of science diplomacy and challenges neocolonial structures by acknowledging the unique lived experiences of local populations. By fostering cross-cultural understanding and inclusivity in research, we can enhance the implementation of FertiQoL and pave the way for novel interventions, increased funding, and policy developments in the Global South, ultimately promoting equitable global health.

## Introduction

Infertility is a global public health issue with severe consequences, such as having a negative impact on Quality of Life (QoL) (1–5). QoL is defined by the World Health Organization (WHO) as “an individual’s perceptions of their position in life in the context of the culture and value systems in which they live, and in relation to their goals, expectations, standards and concerns” (6). According to the WHO, the lifetime incidence of infertility in High Income (HIC) and Low-and Middle-Income Countries (LMICs) is 17.8 and 16.5%, respectively, (7). Despite no significant differences in infertility occurrence between these countries, it is known to pose especially negative outcomes in LMIC populations (8–12). This may be related to the lower prevalence and utilization of infertility services in LMICs due to a shortage of public financing and cultural stigmas associated with publicly disclosing fertility-related issues (13, 14).

Impact on QoL has been used to evaluate personal perception of adjustment to health-related issues and life events (6), as such, the Fertility Quality of Life Tool (FertiQoL) was developed to provide a more standardized way to measure the impact of infertility and involuntary childlessness on QoL (2). Despite being translated into 48 languages and globally validated (15), only a limited number of studies have explored the cultural relevance of FertiQoL items in psychometric testing (16, 17). The current landscape demands a paradigm shift in global health research, necessitating the integration of science diplomacy principles and a heightened focus on cultural sensitivity. Science diplomacy involves the convergence of science, technology, and political interactions to address pressing social, environmental, and economic issues within and between countries, including global public health challenges such as infertility (18, 19). Recent research underscores the importance of context-adapted policies based on decolonized research to effectively tackle global health issues (20). This study seeks to contribute to this shift by examining methodological and cultural considerations in the implementation of the FertiQoL tool in Sudan, thereby aligning with the principles of science diplomacy and challenging neo colonial structures.

Infertility in Sudan presents a significant societal challenge, evident in the increased interest of the Federal Ministry of Health and the rising number of private treatment clinics (21). However, treatments for infertility require individuals to navigate the complex interplay between financial limitations and cultural pressures. The financial burden of infertility treatment prevents healthcare professionals from supporting a majority of infertile individuals. This is because infertility healthcare centers are not equally distributed in developing countries, with more low-income regions depending primarily on limited resources from non-profit organizations who often do not prioritize fertility treatment (22). Thus, the limited access and high cost of treatment is a barrier for many patients. Additionally, rigid gender norms attribute the responsibility for infertility primarily to women. This subjects women to blame and exposes them to societal stigma, often leaving them to seek medical help alone (23, 24). This limited support for infertile women is reflected in studies reporting instances of domestic violence, divorce, and social isolation in LMICs (25). These problems highlight the urgency of addressing infertility in Sudan and other LMICs, with tools like FertiQoL offering insights into understanding this burden.

The current study is part of a larger project examining the feasibility and acceptability of using fertility tools in LMICs, and preliminary results of that project (26–28) indicated that there might be some methodological and cultural considerations that need to be examined further to facilitate implementation of the FertiQoL in Sudan and other LMICs (28). This is a necessary step for research as it positions the Global South at the center to ensure its validity globally. Furthermore, the findings could potentially encourage government funding into medical aid and awareness concerning reproductive health.

One methodological issue that became apparent during the project, was that the mode of administering the FertiQoL might impact how the questions are interpreted and answered. To address this concern, we added an additional dimension to the study to investigate how the mode of administering could impact FertiQoL scores, which, to our knowledge, has not been examined. There is disagreement in the literature about which administration mode of self-assessment enhances accuracy (29–32). Moreover, Likert scales, as used by the FertiQoL, are especially susceptible to these types of response biases, as reported by a study on a Spanish population (33). A global study by Harzing et al. (34) points out that Likert scales lack nuance when it comes to cross-national research due to language biases. For self-assessment of QoL specifically, evidence is mixed, with some studies suggesting that there might not be a difference between face-to-face interviews and surveys (31), while others report that predicting QoL was improved using questionnaire-based than interview-based assessment (30). A recent systematic review on the global use of QoL measures/tools, reported that interviewing is the most commonly used method to assess QoL since it increases response rates and reduces errors due to misunderstanding or confusion about questions (32).

It is well documented that social desirability bias (the desire to present one’s best self) appears more during interviews and other face-to-face interactions than while completing self-administered questionnaires (29, 35). Additionally, acquiescence bias (the propensity toward more ‘yes saying’ and agreeing with others to appear ‘easy going’) is also exaggerated in face-to-face interactions (29, 36). These biases are especially pertinent to sensitive topics or ones that are perceived as violating social norms (36, 37). Characteristics of the interviewer, such as race or gender can also influence the response bias (e.g., social desirability, acquiescence bias) and enhance or degrade the accuracy of the data collected (36). On the other hand, interviewers can pick up on subtle cues, like hesitance that can be indicative of response bias and could potentially mitigate these biases or at least make note of them. It has also been reported that interviews can enhance recall as the interviewer can redirect the interviewee (29).

To examine the impact of the mode of administration we compared provider-administered and participant-completed FertiQoL. We also compared interviews with provider-administered mode (where the provider can clarify items but there is no redirection or prior discussion of anything that could trigger memories) to determine if talking about the issue before completing the FertiQoL (in the interviews) would have an impact. We anticipated that the FertiQoL scores would differ based on mode of administration.

It is also well recognized that the implementation of English language tools can be enhanced by ensuring that translation is augmented with an examination of cultural appropriateness (38, 39). This is exemplified by studies that show that culturally appropriate interventions have better outcomes (40). As the initial development of FertiQoL only sampled participants from countries in the Global North (Australia, Canada, New Zealand, United Kingdom, and the United States), it has proven difficult to directly apply some items of the tool to individuals in LMIC and Global South countries due to differences in culture that mediate the effect of infertility on quality of life in these areas (2, 41). Specifically, there are very few studies assessing the validity of FertiQoL in the African continent, making it difficult to determine exactly which items may be irrelevant or subject to improvement (28). Thus, to understand the importance of cultural appropriateness examination of cultural factors that could potentially impact the implementation of FertiQoL in Sudan is essential.

Some key cultural considerations in this region are the prominent ideology positioning child rearing as a necessity, and the taboo surrounding discussions of infertility (42). This may add pressure for individuals living in this region, making it difficult for them to answer all questions on the tool truthfully. It is therefore necessary to adopt a qualitative approach spearheaded by researchers in the region who have a clear understanding of local culture and societal norms. This could provide a richer insight into the lived experiences of those dealing with infertility as aimed by this study. This is especially important as many studies in the African continent have been conducted by foreign researchers that have not collaborated with local professionals nor taken cultural context into consideration when reporting results, thus perpetuating the cycle of neocolonialism in research (43).

Considering this, the main aim of this mixed methods study was to investigate methodological and cultural factors that could potentially impact the effective implementation of FertiQoL in Sudan and other LMICs, informed by the patients’ perspectives. This approach aligns with the principles of science diplomacy, emphasizing collaboration and inclusivity in research. To achieve this aim, we set out two objectives. The first objective was to determine if FertiQoL scores would differ significantly based on mode of administration (quantitative data). The second objective was to examine whether there were cultural issues that surfaced in the interviews (qualitative data) that could explicate the FertiQoL scores (quantitative data) and vice versa. We used a mixed methods approach because it can help reveal patterns by comparing cases and personal stories from the individuals’ perspectives (44), thereby offering a better understanding of the challenges and opportunities associated with FertiQoL implementation in diverse cultural contexts.

## Materials and methods

### Study design

In this mixed methods study, we used an explanatory sequential design. The study comprised surveys and semi-structured interviews with Sudanese men and women attending a semi-private fertility clinic in Khartoum (capital of Sudan). Participants were randomly assigned to one of the three modalities of administering the FertiQoL: (1) questionnaire completed by participant without assistance of a provider (survey-self-administered); (2) questionnaire completed by a research assistant (survey-provider-administered); and (3) questionnaire completed by the interviewer at the end of the qualitative interview (interview). In the self-administered version, the participants read and answered the items independently. In the provider-administered version, a research assistant read the items and noted the answers. In the interview version, the interviewer conducted the FertiQoL (read the items and noted the answers) at the end of the interview, after the discussion of how infertility has impacted their lives. In both the second and third versions the participants did not see the written FertiQoL. The interviews were used to differentiate the subjective lived experience reported spontaneously (prior to administration of FertiQoL) with that captured by FertiQoL. Additionally, interviewees were asked about their experience using FertiQoL and how they felt about the results (after administration of FertiQoL). At the end of all variations, we debriefed and thanked participants.

### Participants and recruitment

Using convenience sampling we recruited patients attending the clinic from November 2017 until May 2018 and there were no exclusion criteria. This is a tertiary clinic and all patients attending this clinic have been referred here for infertility. Ethical approval was sought and provided by the Universities. Patients were approached in the clinic waiting room by a research assistant and invited to participate in the study, in a private room in the clinic before their appointments (the clinic has long wait times, therefore participants could take part in the study during that wait time). Those who agreed were briefed and signed the consent form. We collected demographic data and administered the FertiQoL for all participants.

We used a sequential random sampling method to include patients to participate in the different arms of the study (self-administered, provider-administered and interview). Eighty-six patients were asked to participate in the survey of which 82 (95.3%) agreed and completed the survey (self and provider-administered). Twenty-two patients were asked to participate in the interview, of which 20 (91%) agreed and completed the interviews. We continued recruitment for the interviews until there was data replication and redundancy, saturation of data and point of diminishing returns was reached (45). The interviews lasted approximately 30 min and the participants were first asked about their demographic information, medical and reproductive history, then open-ended questions about the impact of infertility on their lives, followed by administration of the FertiQoL. We then scored the FertiQoL and discussed those scores (including visual bar charts) with participants. The interviews were audio recorded, transcribed and translated by RRB.

### Materials

Detailed information about the materials used, translation, procedures and data analyses have been published (28). Surveys consisted of a consent form, background information (demographic, medical and reproductive history, e.g., age, past fertility history) and the Arabic FertiQoL. Only the core module of FertiQoL was used, which is scored out of 100, and higher scores indicate higher quality of life. The treatment module was not used as the clinic director suggested that this might make the participants confused about the treatment they will receive (they might think how they respond to the treatment questions will impact on the type of treatment they receive). Interview topic guide contained the same materials as the survey in addition to open-ended questions about the impact of infertility on QoL (e.g., ‘How has infertility affected your life?’) and questions about the FertiQoL results (e.g., after being shown their results, participants are asked: ‘How do these scores compare to your experience, do they accurately reflect what you are feeling?’).

### Data analysis

We used STATA (version 15) to generate descriptive statistics and one-way ANOVA to compare the three groups. We conducted a one-way ANOVA to compare means from the three modes of administering the FertiQoL: self-administered, provider-administered and interview. To estimate the effect size, we calculated Eta-squared (*η*^2^) which indicates the proportion of the total variance in the FertiQoL scores that can be attributed to the mode of administration.

RRB and EK conducted thematic analysis (46), using inductive coding to assess the emerging themes and a second round of thematic analysis to triangulate the data [(44, 47), see (28) for details about the analytic process]. To bolster the trustworthiness of the methods and findings, we used the Critical Appraisal Skills Program best practice guidelines for qualitative research (48, 49).

### Reflexive statement

The research team comprised two seasoned qualitative researchers specializing in infertility. RRB, a clinical psychologist and researcher, brought extensive knowledge of the cultural context (as she is Sudanese) and therefore offered an insider perspective to understanding the data. RRB designed the study, prepared the interview materials, and conducted the interviews. EK, a psychologist with 12 years of experience in infertility, provided insights into the phenomenon and its impact on Quality of Life (QoL). Being non-Sudanese, EK offered an external viewpoint on the cultural aspects. RRB and EK collaborated on qualitative data analysis, result interpretation, and preparing the manuscript. Our awareness of our respective research positions in relation to the content led to insightful discussions during the analysis phase.

## Results

### Socio-demographic and reproductive characteristics

Detailed description of socio-demographic results for the whole sample (*n* = 102) have been published (28). Table 1 shows socio-demographic characteristics separated by mode of administration of FertiQoL. As shown in Table 1, in all three groups most of the participants were women who were educated beyond secondary school and lived in urban areas. Overall, there were no statistically significant differences in the baseline socio-demographic and reproductive characteristics between the three groups. However, there were minor differences between the groups. For example, the average age was different for the three subsamples: in the interview subsample (*n* = 20), the average age was 33 years; in the self-administered questionnaire subsample (*n* = 40), it was 32 years; and in the provider-administered questionnaire subsample (*n* = 42), it was almost 36 years. Most participants in all three groups were infertile for about 4 years and the reason for infertility was ‘female factor only’ in most cases.

**Table 1:** Sample characteristics by mode of administration of the FertiQoL.

Characteristic		Mean (SD)			
		Total (*n* = 102)	Interview (*n* = 20)	Self-administered (*n* = 40)	Provider-administered (*n* = 42)
Age (years)		33.89 (7.82)	32.8 (9.3)	32.4 (3.2)	35.9 (7.5)
Duration of infertility (years)		4.62 (3.46)	4.1 (2.9)	4.3 (3.5)	3.8 (3.2)
		*n* (%)			
Gender	Females	72 (70.6%)	17 (85%)	28 (70%)	27 (64.3%)
	Males	30 (29.4%)	3 (15%)	12 (30%)	15 (35.7%)
Residence	Urban	73 (71.6%)	15 (75%)	27 (67.5%)	31 (73.8%)
	Rural	29 (28.4%)	5 (25%)	13 (32.5%)	11 (26.2%)
Education	Secondary or less	32 (31.4%)	7 (35%)	11 (27.5%)	14 (33.3%)
	More than secondary	70 (68.6%)	13 (65%)	29 (72.5%)	28 (66.7%)
Consanguinity	Yes	54 (52.9%)	11 (55%)	16 (40%)	20 (47.6%)
	No	48 (47.1%)	9 (45%)	24 (60%)	22 (52.4%)
Source of infertility	Female factor only	35 (34.3%)	12 (60%)	7 (17.5%)	16 (38.1%)
	All other sources	67 (65.7%)	8 (40%)	33 (82.5%)	26 (61.9%)
Previous pregnancy (*n* = 72)	Yes	22 (30.6%)	5/17 (29.4%)	10/28 (35.7%)	7/27 (25.9%)
	No	50 (69.4%)	12/17 (70.6%)	18/28 (64.3%)	20/27 (74.1%)

SD, standard deviation; *n*, sample size.

### FertiQoL scores

The mean core score for the sample was (*M* = 76.02, SD = 16.26), the mean domain scores were, emotional (*M* = 71.61, SD = 22.04), relational (*M* = 78.06, SD = 16.62), mind/body (*M* = 74.06, SD = 22.53) and social (*M* = 78.88, SD = 18.24).

### Objective 1: different modes of administration of the FertiQoL

Table 2 shows the FertiQoL core and domain scores (mean and SD) for the three modes of administration. Results of one-way ANOVA showed that there were no statistically significant differences between group means for core FertiQoL scores (*F*(2,99) = 1.58, *p* = 0.21, *η*^2^ = 0.03) and domain scores: emotional (*F*(2,99) = 1.85, *p* = 0.16, *η*^2^ = 0.04); mind/body (*F*(2,99) = 1.95, *p* = 0.15, *η*^2^ = 0.04); relational (*F*(2,99) = 0.18, *p* = 0.83, *η*^2^ = 0.04); and social (*F*(2,99) = 1.67, *p* = 0.19, *η*^2^ = 0.03). These results indicated that there was no significant difference in scores between the three modes of administering the FertiQoL.

**Table 2:** Core and domain score means and standard deviations for the different modes of administration of the FertiQoL in this sample of Sudanese men and women (*n* = 102).

FertiQoL	Mean (SD)		
	Interview *n* = 20	Self-administered *n* = 40	Provider-administered *n* = 42
Core	76.82 (11.41)	72.60 (19.21)	78.89 (14.84)
Emotional domain	66.67 (17.83)	68.96 (23.61)	76.49 (21.81)
Relational domain	80.00 (10.87)	77.92 (15.63)	77.27 (19.78)
Social domain	81.46 (12.64)	74.79 (22.49)	81.55 (15.43)
Mind/body domain	79.17 (16.17)	68.75 (27.86)	76.68 (18.54)

SD, standard deviation; *n*, sample size.

### Objective 2: thematic analysis and triangulation

Two main themes emerged from thematic analysis of the interview data, with several subthemes. Theme 1: Lived experiences and FertiQoL scores, Subtheme (1.a) FertiQoL confirmed the lived experience for the majority of participants; Subtheme (1.b) discrepancy between the lived experience reported in the interviews and the participants’ FertiQoL scores for some participants. Theme 2: Potential utility and acceptance of FertiQoL, Subtheme (2.a) FertiQoL represented an icebreaker to start sensitive conversations; Subtheme (2.b) Clarifications for specific items (FertiQoL questions); and Subtheme (2.c) Other feedback regarding implementation of the FertiQoL in Sudan; (See Table 3 for these themes, with descriptions and explanations of the themes).

**Table 3:** The themes that emerged from the data with the description and explanation of themes.

Themes	Description	Explanation
**Theme 1:** Lived experiences and FertiQoL scores	This theme explores participants’ reactions to FertiQoL results, focusing on whether they agreed with the scores presented to them.	While majority of participants self-reported lived experiences as shared in interviews was consistent with FertiQoL scores, inconsistencies existed for some participants.
**Subtheme 1.a:** FertiQoL confirmed lived experience for majority of participants	This subtheme explores instances where participants’ FertiQoL scores were reflective of their lived experiences as reported in interviews.	The majority of participants acknowledged that FertiQoL accurately reflected their emotional experiences related to infertility.
**Subtheme 1.b:** Discrepancy between lived experience reported in interviews and participants’ FertiQoL scores for some participants	This subtheme explores instances where participants’ self-reported experiences in interviews did not align with their FertiQoL scores, highlighting potential discrepancies.	Despite congruence for most participants, some showed hesitancy or denial in reporting negative impacts during interviews, which contradicted their FertiQoL scores.
**Theme 2:** Potential utility and acceptance of FertiQoL	This theme explores participants’ perspectives on how FertiQoL could extend beyond its intended purpose, potentially serving as an icebreaker or offering insights into financial aspects.	FertiQoL emerged as a self-reported suitable tool by the majority of participants, but some reported shortcomings such as the lack of items related to financial considerations.
**Subtheme 2.a:** FertiQoL represented an icebreaker to start sensitive conversations	This subtheme focuses specifically on the potential of FertiQoL to initiate conversations about the impact of infertility, serving as a cue for individuals to reflect on their experiences.	Participants highlighted that FertiQoL could serve as an icebreaker, prompting reflection on the broader impact of infertility and providing patients with a language to articulate their experiences. They also pointed out the absence of financial considerations in the tool, suggesting its potential enhancement for a more comprehensive assessment.
**Subtheme 2.b:** Clarifications for specific items (FertiQoL questions)	This subtheme delves into participants seeking clarifications for certain FertiQoL items, shedding light on potential issues related to the comprehensibility of these questions.	Issues arose regarding the clarity of specific FertiQoL items, as evidenced by instances where participants sought clarifications.
**Subtheme 2.c:** Other feedback regarding implementation of FertiQoL tool in Sudan	This subtheme delves into participants’ feedback regarding the suitability of FertiQoL in Sudan.	While the majority found FertiQoL suitable, others pointed out the absence of financial considerations. This suggests that modifications may be needed to enhance the tool’s comprehensiveness.

### Theme 1: lived experiences and FertiQoL scores

This theme encompassed whether the FertiQoL confirmed the lived experience or if there were discrepancies between FertiQoL scores and the lived experience.

#### Subtheme (1.a): FertiQoL confirmed the lived experience for the majority of participants

The majority of participants expressed agreement with the FertiQoL results presented to them at the end of the interview, affirming that the scores resonated with their personal experiences. In addition, triangulation of data indicated that for most participants the FertiQoL results (quantitative data) were congruent with their reported lived experience (qualitative data). For example, PT 2, a 33-year-old man who had one of the lowest scores on the Emotional domain (33.3) stated: “it’s been five years and God has not given me a child, and this is what’s affecting me, a lot for me, it’s really hard for me, it has affected all aspects!”

#### Subtheme (1.b): discrepancy between the lived experience reported in the interviews and the participants’ FertiQoL scores for some participants

Despite the congruence between the FertiQoL and the reported lived experience for most participants, we found that some of the participants either denied or were hesitant about reporting a negative impact of infertility in response to the open-ended questions in the interviews. However, this negative impact was evident from their responses on the FertiQoL, especially on the emotional and relational domains. For example, PT 5, a 41-year-old woman, when asked how infertility had affected her life in an open-ended.

question, she denied relational problems and upon further probing when asked specifically if her infertility had affected her married life she responded: “no it has not affected me.” However, her relational domain score was only slightly above average (62.5), suggesting some impact on the spousal relationship. Furthermore, in most of these cases when the participants were shown their FertiQoL results at the end of the interview, they confirmed that they agreed with the scores. For example, when we asked PT 3, a 31-year-old woman, if her life had been impacted by infertility in the interview prior to the FertiQoL, she stated: “no it has not been affected, I do not feel it has been affected!” However, after seeing that her FertiQoL emotional score was 62, she said “yes, there is a very small effect.”

Another area where there was a difference between the qualitative and quantitative data was social pressure. Social pressure was a pervasive theme that emerged in the qualitative analysis, it was discussed by 12 of the 17 women interviewed. Many of the participants described social pressure as a negative consequence of infertility, especially in the form of intrusive questions from family, friends and society in general. Triangulation of the data revealed that the codes for this theme all related to being asked too many questions about childlessness by family and friends and the pressure this creates, while the FertiQoL social domain items mainly pertain to social engagement and social support, except for item 22 which asks directly about social pressure. Qualitative data analysis also indicated that the social domain score was the highest (compared with emotional, relational and mind/body) domain score for the whole sample.

An example of this triangulation includes PT 20, a 25-year-old woman. When asked about the impact of infertility on her life, she said: “with my husband I have no problem, but with social relationships, whenever I meet someone, they always ask ‘not three yet? What’s happening? What have you done so far [regarding treatment]’ they always ask questions” and when asked how that makes her feel she replied: “I feel upset, I feel irritated!”

However, this was not reflected on her FertiQoL scores, core (80.2) and social domain (91.7). A similar situation occurred with PT 13, a 24-year-old woman (Social domain score 87.5) who said “…of course, you know I mean here in Sudan you know this issue of having kids is important. You get married they ask you ‘not three yet?’ The issue, it has an effect on your emotions even on your husband, his friends ask him ‘not three yet?’ So, the emotional effect is always huge. I cannot tell you, with every period you have this emotional reaction and disturbance, just this awful thing.” From this statement one would conclude that infertility is affecting her socially and emotionally. However, her FertiQoL score for the social domain (88) does not reflect this accurately, but her emotional domain score (66) was more consistent with what she stated. In addition, several women reported pressure from family and friends asking questions but when asked directly if this pressure would impact social engagement, they said no.

### Theme 2: potential utility and acceptance of FertiQoL

This theme was about the different participants’ experience and feedback which demonstrated that FertiQoL could potentially be used beyond its designed purpose, in different cultural contexts. There were three subthemes:

#### Subtheme (2.a): FertiQoL represented an icebreaker to start sensitive conversations

We found that the FertiQoL can potentially be used as an icebreaker or a ‘cue’ for patients to think about the impact of infertility on their lives as it provides the language to describe their experiences. This was especially evident from the shift in attitude that some participants had when they were shown their results at the end of the interview. Therefore, it can potentially be used by individuals as a reflective tool throughout their infertility journey to enable them to think about how infertility has impacted their lives. For example, PT 11, a 27-year-old woman, described being very upset by her infertility in the interview, but when shown that her score for the emotional domain was high (50), she reflected “I’m under pressure from these things. So perhaps the pressure I’m under is less when I’m undergoing treatment.”

#### Subtheme (2.b): clarifications for specific items (FertiQoL questions)

This theme encompassed issues regarding the comprehensibility of some of the FertiQoL items. Items were included in this theme only if more than one respondent sought clarification for the item. Table 4 shows the six items where the participant either asked what the item meant, looked confused or was unable to answer. For example, ‘Q15-Have fertility problems strengthened your commitment to your partner?’, required clarification for five participants, mainly because the connection between the concepts ‘commitment’ and ‘fertility problems’ was unclear. For details regarding the items (English and Arabic) as well as how many participants sought clarification for each item see Table 4 and supplemental materials for full versions of English and Arabic FertiQoL.

**Table 4:** English and Arabic versions of the FertiQoL items that needed clarification and the number of participants who sought clarification.

FertiQoL item number	English version	Arabic version	Number of participants who sought clarification
Q4	Do you feel able to cope with your fertility problems?	هل تشعر بأنك قادرا على مواجهة مشاكلك الناجمة عن الخصوبة؟	2
Q7	Do your fertility problems cause feelings of jealousy and resentment?	هل تسبب مشاآل الخصوبة لديك الشعور بالغيرة و الاستياء؟	3
Q9	Do you fluctuate between hope and despair because of fertility problems?	هل تتأرجح مشاعرك ما بين الأمل واليأس بسبب مشاكل الخصوبة؟	4
Q10	Are you socially isolated because of fertility problems?	هل أنت معزول اجتماعيا بسبب مشاكل الخصوبة؟	2
Q15	Have fertility problems strengthened your commitment to your partner?	هل عززت مشاكل الخصوبة لديك التزامك لشريك حياتك؟	5
Q22	Do you feel social pressure on you to have (or have more) children?	هل تشعر بضغط اجتماعي عليك لأن يكون لديك أطفال (أو المزيد من الأطفال)؟	2

Q, question.

#### Subtheme (2.c): other feedback regarding implementation of the FertiQoL in Sudan

Most participants felt it was suitable (28), however, one participant, PT 13, a 24-year-old woman, felt that it was missing the financial aspect. After viewing her scores, she said that it should be lower “because the impact on my life is more, I mean from a financial perspective, from the treatment that could be very expensive.” And when asked if the tool was comprehensive, she said “only the financial was not mentioned, you talked about everything…except this financial, it really does have a big impact…if you included the financial it would give you a better score, more accurate.”

## Discussion

In line with previous research, the results indicated cultural factors that could potentially impact the implementation of FertiQoL (42, 50), however, methodologically, the different modes of administration of the tool appeared to be comparable in this context. The results overall suggested that participants perceived the FertiQoL to be comprehensive and understandable and had utility beyond assessment of fertility related QoL such as helping patients reflect on how infertility had affected their lives, which might be especially relevant for cultures where such discussions are difficult (50). It became evident that the interpretation of certain items or domains could benefit from a culturally sensitive perspective to align more closely with patients’ lived experiences. Addressing these cultural considerations could enhance the implementation of FertiQoL, aligning with Khupe and Keane’s assertion that research should be culturally driven and based on their culture, language, and ideology to be advantageous to people (51). This can only be accomplished through the removal of Eurocentric perspectives from research to make it relevant to the Global South.

Specifically, interpretation of quantitative results indicated that FertiQoL mean scores overall and in each domain were not affected by mode of administration of the FertiQoL, consistent with published reviews and studies regarding mode of administration of surveys and questionnaires (35, 52, 53). This could be because the participants were equally forthcoming (or not) while addressing the items in all three modalities. It appears that social desirability, which is related to worry about creating bad impressions, is equally applicable to the different modalities of survey (interview, self-administered focus group etc.), since they all rely on self-report (35, 52–54). However, one caveat is that these subsamples appeared to be demographically and medically similar therefore the same might not be true for groups that are very different from each other.

The themes that emerged indicated that for the most part the FertiQoL confirmed the lived experience as expressed in the interviews except for a few minor discrepancies due in large part to cultural nuances. These discrepancies would typically be disregarded in neocolonial research, but the current study’s decolonised approach puts these details in the foreground. It also appeared that the FertiQoL is a comprehensive tool from the patient’s perspective, except for the lack of impact of financial burden of infertility, which might be especially significant in places like Sudan where the cost of treatment is not covered by the government (24). This is an important consideration as women with lower incomes rely more on reproductive health interventions from the government than women with higher income (55). Potential utility and extended use of the tool emerged from the analysis as FertiQoL appeared to help participants see that there is an impact and which areas were affected, confirming that it can be used by providers to help patients identify problem areas and self-reflect. The participant’s emphasis on the financial burden of treatment can contribute to the science diplomacy discourse by shedding light on this issue for infertile individuals. A systematic review by Njagi et al. (14) was the first to compare medical costs for Assisted Reproductive Technology (ART) across LMICs and found them to be up to 200% of the GDP per capita in Africa and South-East Asia. Whereas the lower costs of ART in Eastern Mediterranean regions and Americas was correlated with regulatory policies of ART and more government funding mechanisms. The present findings may encourage further investigation of these financial issues specifically in Sudan and similar Global South countries and promote the development of appropriate ART regulations and public funding mechanisms for infertility treatment. This can ensure wider access by acknowledging and integrating infertility treatment in universal healthcare funding coverage. Outcomes from science diplomacy research have already come into fruition as evidenced by Gerrits et al. (13) who set up a project funded by the Dutch Ministry of Foreign Affairs to increase infertility awareness in Kenya and Ghana. Organizations such as this could foster improved diplomatic relations amongst Global South and Global North regions and motivate increased financial support for underfunded public health issues.

Furthermore, thematic analysis helped identify specific items that required clarification. This also highlights the aforementioned importance of verifying research tools’ effectiveness in the Global South by taking into account the existing cultural and linguistic differences. For the item that asks about ‘coping’ with the fertility problem, it appeared that the translation of the term ‘coping’ might be the source of confusion. In the Arabic FertiQoL the word used for coping is ‘muagahat (مواجهة)’, which can mean to ‘cope with’ but translates more closely to ‘face’ or ‘confront’ (56). In the item that asks about whether the person fluctuates between hope and despair, it appeared that the use of the Likert scale for this item might have been confusing because there seem to be two dimensions (the hope-despair dimension and the agree-disagree dimension), making it difficult to know how to complete this item. For the other four items that required clarification, it seemed more likely that the cultural or social context for the items might differ depending on where the FertiQoL is used. For example, the item about social isolation, might be difficult to interpret within the Sudanese context, where social engagements are obligatory and not attending them would be frowned upon, therefore the option to socially isolate is not available nor would someone be socially isolated by the society. It could also be the case that the ability to disclose about the topic might make one appear inferior, e.g., the item about jealousy might not be answered honestly as jealousy is perceived as a very negative attribute in Arab cultures because it is associated with the concept of the ‘evil eye’ which is perceived as very harmful (57) and sometimes the *cause* of infertility (58).

The presence of such factors highlights the importance of researchers who are well acquainted with the cultural context in which the study is being conducted. For instance, it is generally well known that most countries in the Global North tend to have individualistic and autonomic structures, whereas most countries in the Global South have more collectivist tendencies (59). This makes it clear that results from the Global North, where most research is typically conducted, cannot be generalized to the rest of the world population (60). In line with this, this study underscores specifically how these social nuances affecting participants’ interaction with the FQoL questionnaire can be different from participants from Global North countries. Results from such studies may help further science diplomacy within the Global South through South–South cooperation, where developing countries share resources and expertise in the process of meeting development goals via joint efforts (61). Past policy recommendations from the Task Team for South–South Cooperation (TT-SSC) include the improvement of information quality to align more closely with national systems, as well as the implementation of evidence-based knowledge sharing between countries to better address their needs. This has been crucial in the management of Global South health issues such as the Ebola virus outbreak in the past (61, 62). The specific focus of the study on the social, cultural and financial domains of infertility in Sudan and by extension, other similar countries in the Global South, may foster a more holistic understanding of the challenges as well as methods of implementing infertility services throughout the region (13, 61). Additionally, the observations from this study could only be made due to the employment of mixed methods. An exclusively quantitative study would not have given participants the opportunity to elaborate on their doubts regarding the questionnaire and general thoughts on their perceived quality of life.

We anticipated that the importance of having offspring to fulfill the goal of forming a family would lead to a significant impact on the social aspect of the individual’s life. However, triangulation of the data revealed that while social pressure was reported by many participants as the main negative consequence of infertility, the overall social domain score was the highest domain score for the sample. An examination of this discrepancy indicated that it could be because items in the social domain in the FertiQoL are focused on social support and engagement more than social pressure and since all items are given equal weight in the score this might have dwarfed the impact of social pressure. Furthermore, social support and engagement might not be affected by social pressure in Sudan as they are woven into the cultural fabric of Sudan and other collectivist societies (63–65). As the use of the FertiQoL continues to grow in collectivist countries like Turkey, Iran, India, South Africa, China, Korea, and Japan (66–73), interpreting the social domain scores within the cultural context becomes more important and clinicians and researchers should be aware of this issue. Ways to better understand the social domain within a cross-cultural context should be examined in future research using methodology targeted at improving questionnaire design, such as cognitive interviewing, which has proven to be effective across cultures (74).

Despite the methodological and cultural considerations, results in general indicated that FertiQoL is a useful disease-specific QoL measure that can help express the effect of disease on QoL (part of burden of disease) from the patient’s perspective (5). This is important because understanding and estimating the burden of disease can precipitate action to reduce it (75). Thus, as previously mentioned, this tool may be essential when justifying the need for financial investments by stakeholders into research examining prevention and treatment of infertility. A global study by Zhang et al. (76) noted that in instances where medical insurance for infertility is offered, patients are more likely to seek treatment which may eventually improve their quality of life. While providing these treatments and making policies to avoid infertility may be costly, research estimated that these investments may pay off (14, 77). For instance, it has been estimated that public investments into technologies for *in-vitro* fertilization (IVF) can result in future economic benefits for the South African government due to the potential increase in tax-payer citizens, maintenance of a healthy and sustainable workforce, and reducing long-term healthcare costs, among other reasons (77). This is significant for the economy for LMICs like Sudan where financial costs of IVF treatments are unaffordable for many patients who may need it (14).

### Strengths and limitations

The most important limitation of the current study was the use of a small convenience sample that was largely homogeneous and consisted mostly of educated women at a fertility clinic in Khartoum (capital of Sudan), which limited the generalizability of the results. This might not be problematic since FertiQoL has been validated in several studies with much larger samples (15) and generalizability was not the purpose of this study. Rather, the current study was conducted to examine the patients’ perspectives on the FertiQoL using a mixed methods design where a small sample size would have less impact on results (78).

The key strength of the study was the application of a mixed methods approach, which enhanced the rigor of the study (79). Mixed methods techniques such as triangulation could increase the validity of the results since they help integrate qualitative data to provide potential explanations for the quantitative data (80). This was important because to our knowledge, the FertiQoL has not been studied qualitatively. Therefore, our results could help shed light on issues that could have otherwise been overlooked (80). This includes the cultural underpinnings behind the lived experiences of participants, which may be unique to those living in the Global South. This indicates the importance of mixed methods studies, especially within this region, as statistical data can often be fragmented or poorly analyzed without context (81). The second strength was the adherence to best practices guidelines of qualitative analysis (46, 48, 49). The final strength was that the research team was multinational and multidisciplinary, with the specific culture in question being represented by the primary author (RRB). This provided the appropriate cultural lens with which to approach the study and participants being interviewed (82), which in turn led to a thorough knowledge and understanding of the subject, cultural considerations, and the methodology, ultimately increasing trustworthiness of the results.

Since this was a small homogenous sample, future research should focus on replicating this study with larger and more diverse samples that include more men, people in rural areas and less educated individuals as well as community based non-clinical samples.

## Conclusion

The main finding of the study is that FertiQoL, a tool designed to assess fertility-related quality of life, is perceived as comprehensive and understandable by participants. The results indicate that the tool has utility beyond assessing fertility-related quality of life, as it helps patients reflect on how infertility has affected their lives. The study emphasizes the importance of considering cultural factors in the implementation and interpretation of FertiQoL. Specific cultural nuances, such as the impact of financial burden on infertility, were highlighted, underlining the need for a culturally sensitive perspective in utilizing the tool. The study also reveals discrepancies in the interpretation of certain items due to cultural and social contexts. Despite these considerations, FertiQoL emerges as a valuable disease-specific quality of life measure that can aid in expressing the impact of infertility on patients’ lives and as a flexible model that can be adapted to diverse cultural contexts. The findings suggest that the tool has potential utility for healthcare providers in helping patients identify problem areas and engage in self-reflection. The study contributes to the understanding of how cultural factors can influence the application and effectiveness of health-related research tools, particularly in the context of infertility in the Global South.

In conclusion, our study on the implementation of FertiQoL has revealed promising insights that align with the goals of science diplomacy. As evidenced by the cultural factors influencing the tool’s implementation, our findings underscore the significance of embracing cultural sensitivity in shaping public health policies and interventions. A key takeaway from our research is the importance of recognizing and addressing neocolonial structures in public health research. Our approach highlights the need to remove Eurocentric perspectives, making research more relevant to the Global South. As we move forward, it is imperative to consider the impact of local culture and language on the interpretation of research tools, reinforcing the idea that successful science diplomacy should be driven by the culture, language, and ideologies of the communities it aims to serve. As we extend our gaze beyond the horizons of South America and Asia, our findings emphasize the need for more research initiatives in regions such as the Middle East and Africa. By doing so, we can further incorporate decolonial thinking and heritage diplomacy into the discourse surrounding science diplomacy. Our study, situated within a multicultural and multidisciplinary framework, supports the call for evidence that transcends geographical boundaries, acknowledging researchers as protagonists irrespective of state hierarchies. Looking ahead, we propose that the lessons learned from the implementation of FertiQoL serve as a foundation for further research endeavors in underrepresented regions.

## Data Availability

The raw data supporting the conclusions of this article will be made available by the authors, without undue reservation.
